# MUCL1 in triple-negative breast cancer: a novel marker associated with the luminal androgen receptor subtype

**DOI:** 10.1186/s13058-026-02283-y

**Published:** 2026-04-15

**Authors:** Franz-Leonard Klaus, Isabella Kitzke, Evelyn Klein, Carolin Mogler, Alexander Muckenhuber

**Affiliations:** 1 Institute of Pathology, TUM School of Medicine and Health, TUM, Munich, Germany; 2 Department of Gynecology and Obstetrics, TUM School of Medicine and Health, TUM University Hospital, Klinikum Rechts Der Isar, Munich, Germany

## Abstract

Triple-negative breast cancer (TNBC) comprises several molecularly distinct subtypes. The luminal androgen receptor (LAR) subtype is characterized by AR expression, displays luminal-like gene signatures, and often co-expresses FOXA1 and GCDFP-15, but its immunohistochemical identification remains challenging. Through transcriptomic analyses of GEO datasets, we identified MUCL1 as a candidate marker and validated its protein expression in a retrospective cohort of 106 TNBCs. MUCL1 was expressed in 25% of cases in our cohort, enriched in apocrine and lobular carcinomas, and strongly correlated with AR, FOXA1, and GCDFP-15. MUCL1-positive tumors occurred in older patients, were larger, and showed lower grade and proliferation, features which are known to be associated with the LAR subtype. Importantly, MUCL1–/AR+ tumors resembled MUCL1–/AR– rather than MUCL1-positive cases, suggesting that AR expression alone may not fully capture the underlying biology of this subgroup, while MUCL1 expression was associated with a more coherent clinicopathological phenotype aligned with LAR-associated features. Transcriptomic enrichment of hormone response and metabolic pathways in MUCL1-high tumors supports this association. Evidence linking both LAR and MUCL1 to reduced chemotherapy sensitivity highlights the clinical relevance of MUCL1.

**Supplementary Information:**

The online version contains supplementary material available at 10.1186/s13058-026-02283-y.

## Introduction

Triple-negative breast cancer (TNBC) is a biologically diverse and clinically often aggressive subtype of breast cancer defined by the absence of estrogen receptor (ER), progesterone receptor (PR), and HER2 expression. TNBC accounts for approximately 15–20% of all breast cancers and is associated with early onset, higher histological grade, and a greater risk of recurrence and metastasis compared to other molecular subtypes [1–3]. As established targeted therapies used in other breast cancer subtypes are ineffective in TNBC, chemotherapy remains the primary treatment; however, responses are often suboptimal and outcomes remain poor [4].

Transcriptomic profiling has revealed considerable heterogeneity within TNBC, leading to the identification of distinct molecular subtypes, including basal-like, mesenchymal, immunomodulatory, and the luminal androgen receptor (LAR) subtype. The LAR subtype comprises a smaller fraction of TNBCs (~10–15%) and is characterized by androgen receptor (AR) expression, luminal-like gene signatures, and frequent apocrine histological features, therefore often referred to as the molecular apocrine subtype [5–7]. Although these tumors often exhibit lower proliferative indices and may be sensitive to anti-androgen therapies in experimental settings, their identification in clinical practice remains challenging [8, 9]. AR immunohistochemistry is commonly used to identify LAR tumors, yet AR expression alone lacks specificity in TNBC, and it is also frequently observed in ER-positive luminal cancers [10, 11].

Markers such as FOXA1 and GCDFP-15 (prolactin-induced protein) have been proposed to capture the molecular apocrine phenotype. FOXA1 is a pioneer transcription factor that facilitates AR chromatin binding, while GCDFP-15 is a downstream AR-regulated secretory protein and a classic marker of apocrine differentiation [12–14]. GCDFP-15 is frequently expressed in both morphologic apocrine carcinomas and TNBCs with a LAR-associated gene expression profile, with reported positivity rates of approximately 70–75%. However, despite its relatively high sensitivity, its specificity for the LAR subtype is limited, as expression can also occur in non-LAR tumors [7, 13, 15]. Likewise, FOXA1 is broadly expressed across luminal breast cancers and is not exclusive to AR-positive TNBC [16]. These limitations underscore the need for additional, more specific biomarkers that can reliably distinguish molecular apocrine TNBC and guide subtype-specific classification and treatment strategies.

In an exploratory analysis of publicly available gene expression datasets (GEO), we identified MUCL1 (also known as Small Breast Epithelial Mucin, SBEM) as a gene with elevated expression in a subset of AR-positive TNBCs. MUCL1 encodes a mucin-like glycoprotein with highly restricted expression in normal breast epithelium, breast cancer and limited expression in other tissues [17]. It is associated with chemoresistance and poorer survival in TNBC and it has been linked to tumor cell motility and epithelial–mesenchymal transition (EMT), supporting its relevance as a potential oncogenic effector [18, 19]. Although previously implicated in HER2-positive and ER-negative breast cancers [20], its role in the AR-positive (LAR) subtype of TNBC has not been systematically investigated.

In this study, we analyzed MUCL1 protein expression by immunohistochemistry in a well-characterized retrospective cohort of TNBC cases. Our objectives were to (i) determine the frequency and pattern of MUCL1 expression, (ii) assess its association with AR status and clinicopathologic features, and (iii) evaluate its potential as a diagnostic and prognostic biomarker in molecular apocrine TNBC. Our findings identify MUCL1 as a previously unrecognized marker enriched in AR-positive TNBCs, with potential diagnostic and therapeutic relevance for the LAR subtype.

## Material and methods

### Marker identification (in silico analysis)

#### Datasets and signature derivation

To identify candidate markers of the luminal androgen receptor (LAR) subtype, we analyzed two publicly available microarray datasets from the Gene Expression Omnibus (GEO): GSE1561 and GSE68892. Both datasets were generated using the Affymetrix Human Genome U133A Array platform (GPL96) and include breast cancer cases, among them tumors classified as LAR subtype based on transcriptomic profiling.

In GSE1561, six LAR cases were identified among a total of 49 breast tumors, which included both triple-negative and hormone receptor-positive cases. Differential gene expression analysis was performed by comparing LAR tumors to non-LAR tumors.

In GSE68892, ten LAR tumors were identified among 99 breast cancer cases, comprising 41 triple-negative and 58 ER- and/or PR-positive tumors. Here, differentially expressed genes were obtained by comparing the LAR cases to the non-LAR triple-negative subset.

DEG analysis was performed using the limma R package, with statistical significance defined as an adjusted *p*-value < 0.01 and an absolute log2 fold change > 0.5. Only genes that were significantly and concordantly regulated in both datasets were retained for downstream analysis. This intersection yielded a 46-gene LAR signature, including both upregulated and downregulated genes. These were subsequently used for scoring and classification of LAR-like tumors. The full gene list is provided in the Supplemental Material.

#### Application to GSE137356

The LAR signature was applied to an independent TNBC dataset, GSE137356, which comprises 381 tumor samples profiled on the Almac Diagnostics Custom Xcel Array platform (ADXECv1a520743). Because this array differs from the original Affymetrix U133A platform, gene identifiers were manually mapped across platforms. Of the 46 signature genes, 45 could be mapped. For each gene, the probe (Spot ID) was empirically selected based on optimal expression characteristics across the dataset.

To quantify the LAR phenotype in each tumor, a LAR score was calculated as the sum of normalized expression values of positively associated genes minus the sum of negatively associated genes. Score computation was performed in R, using base functions without additional scoring packages.

Based on the resulting score distribution, tumors in GSE137356 were empirically stratified into LAR score-high and LAR score-low groups (cut-off 190), allowing for clear separation of phenotypes (see Supplementary Fig. 4 for score distribution). Differential gene expression analysis was then performed between these two groups to further characterize transcriptional differences associated with the LAR-like phenotype. The full list of differentially expressed probe sets is provided in the Supplemental Material.

#### Gene set enrichment analysis (GSEA)

To explore transcriptional programs associated with MUCL1 expression, we performed Gene Set Enrichment Analysis (GSEA) in the GSE137356 dataset. Tumors were stratified into MUCL1-high and MUCL1-low groups using an empirically determined cutoff of 7.5.

GSEA was conducted using the fgsea R package and the Hallmark gene sets from the Molecular Signatures Database (MSigDB). Enriched pathways were identified based on adjusted p-values and normalized enrichment scores (NES).

### Patients and tumor samples

The study cohort consisted of 106 female patients diagnosed with triple-negative breast cancer. The histological subtypes included apocrine (n = 6), lobular (n = 6), medullary (n = 8), metaplastic carcinoma (n = 12), and breast cancer of no special type (n = 74). Formalin-fixed, paraffin-embedded (FFPE) breast cancer tissue samples were used for the determination of estrogen receptor (ER), progesterone receptor (PR), and HER2 status. Additionally, the Ki-67 proliferation index was evaluated. Clinico-pathological and demographic data were obtained from clinical databases, pathological reports, and the Bavarian Cancer Registry. Tumor histology and grading were classified according to the 2019 WHO Classification of Tumors of the Breast. Apocrine morphology was defined based on typical cytological features, namely abundant eosinophilic granular cytoplasm with distinct cell borders and prominent nucleoli. All patients underwent curative surgery at the Interdisciplinary Breast Center of Klinikum rechts der Isar, Technical University of Munich (TUM). Treatment recommendations were formulated through case discussions at an interdisciplinary tumor board.

### TMA

In this study, a tissue microarray (TMA) was constructed using tumor samples with a core diameter of 2 mm, with samples represented in triplicates. The TMA construction was performed using the TMA Grand Master device (3DHISTECH). Tumor regions were carefully annotated on both the histological slides and the corresponding tissue blocks by expert gynecopathologists (F.-L.K., A.M.) to ensure accurate sampling. Tumor samples described in the patient and tumor sample section were included in the TMA. Kidney tissue served as an internal control. The resulting TMAs were used for subsequent immunohistochemical analyses.

### Immunohistochemistry

Immunohistochemical staining was performed on TMA sections from archival breast cancer resection specimens. Antibodies and staining specifications, including clone, supplier, and dilution, are listed in the Supplementary Material.

All stainings were carried out using an automated immunostaining system (BenchMark XT, Ventana Medical Systems, Tucson, AZ, USA) with 3,3′-diaminobenzidine (DAB) as the chromogen and the ultraView detection system.

Ki-67 was evaluated according to the recommendations of the International Ki-67 in Breast Cancer Working Group. For AR and FOXA1, immunoreactivity was quantified using the H-score. For categorical analyses, AR expression was considered positive if the H-score was ≥ 1. This cutoff was chosen in line with previous studies, as summarized by a recent meta-analysis, which reported that thresholds of ≥ 1% and ≥ 10% nuclear positivity are most commonly applied in the literature [21]. For MUCL1, GCDFP-15, CLCA2, SPINK8, CYP4F8, ABCC11, and SOX10, a product score was used, combining staining intensity (0 = negative, 1 = low, 2 = moderate, 3 = high) and the percentage of positive tumor cells (0 =  < 1%, 1 = 1–10%, 2 = 11–50%, 3 = 51–100%). AURKA expression was assessed by estimating the percentage of positive tumor cells in hotspot areas. CK18 staining was evaluated using an intensity score from 0 to 3 (negative to high). For RB1, nuclear staining was scored as follows: 0 =  < 1%, 1 = 1–10%, 2 = 11–50%, 3 =  > 50%.

For categorical analyses, MUCL1 expression was classified as negative (score 0), low (scores 1–4) or high (scores 6 or 9). For CLCA2, SPINK8, CYP4F8, and ABCC11, low expression was defined as scores 0–4 and high expression as scores 6 or 9.

All immunohistochemical evaluations were performed by expert gynecopathologists.

### Statistical analysis

All statistical analyses were performed using R software (version 4.4.1).

Categorical clinicopathological variables (e.g., tumor stage, subtype) were compared between MUCL1-defined subgroups using Pearson’s chi-square test. Continuous variables (e.g., AURKA, Ki-67, age) were assessed for normality and analyzed using either Student’s t-test or the Wilcoxon rank-sum test, as appropriate.

The Wilcoxon test was also applied for comparisons of ordinal variables between two groups (e.g., GCDFP-15 scores). For comparisons across more than two groups (e.g., MUCL1-negative, -low, and -high), the Kruskal–Wallis test was used.

Correlations between continuous or ordinal marker expression levels (e.g., AR, FOXA1) were evaluated using Spearman’s rank correlation coefficient.

All p-values were two-sided, and a significance threshold of *p* < 0.05 was used throughout.

### Use of artificial intelligence tools

ChatGPT (OpenAI, San Francisco, USA) was used to assist with language refinement of the manuscript. All scientific content was written, verified, and approved by the authors.

## Results

### Identification of MUCL1 as a defining transcriptomic feature of LAR-TNBC

**Fig. 1:**
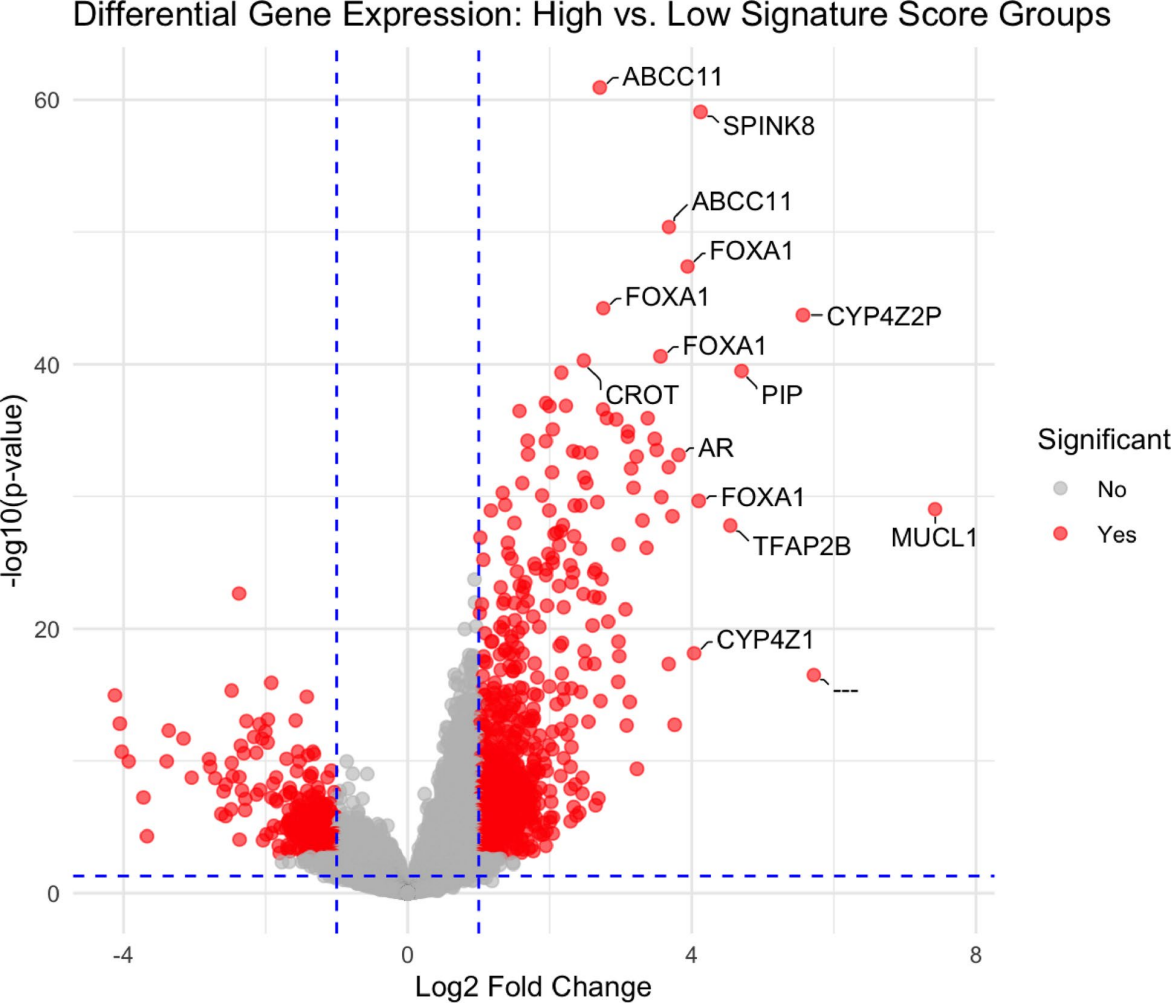
Volcano plot showing differentially expressed genes between tumors with high and low luminal apocrine scores. Red dots indicate significantly regulated genes (adjusted *p* < 0.05, |logFC|> 1). Dashed blue lines mark the thresholds for fold change and nominal significance. Gene symbols were labeled if they met *p* < 1 × 10⁻^40^ or logFC > 3.8. Lines connect labels to their respective data points

To identify candidate genes defining the luminal-apocrine (LAR) subtype of triple-negative breast cancer (TNBC), we analyzed transcriptomic data from GSE137356. Differential gene expression analysis between tumors with high and low luminal apocrine scores revealed a distinct transcriptional separation (Fig. 1). The majority of highly upregulated genes in high-score tumors included *MUCL1, PIP, ABCC11, SPINK8, CYP4Z2P*, and multiple probe sets for *FOXA1 as well as AR*, reflecting a pronounced LAR-associated transcriptional program. Among these genes, *MUCL1* showed the highest log-fold change in LAR-high tumors and was therefore selected for further immunohistochemical evaluation.

**Fig. 2:**
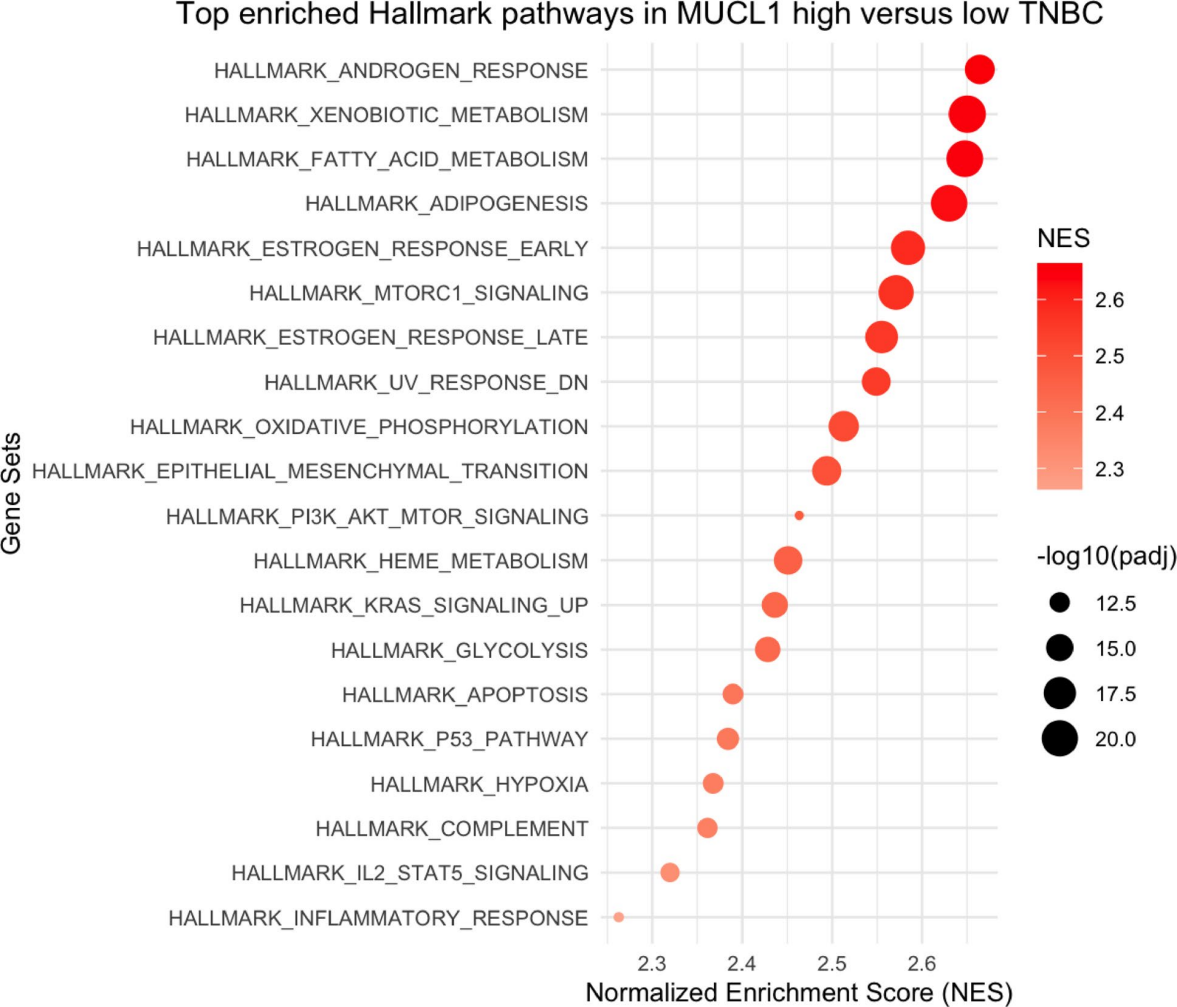
Top enriched Hallmark gene sets in MUCL1-high versus MUCL1-low TNBC tumors. Gene set enrichment analysis (GSEA) was performed on the GSE137356 dataset using the MSigDB Hallmark collection. The dot plot shows the top 20 enriched pathways ranked by normalized enrichment score (NES). Dot size corresponds to –log_10_ (adjusted *p*-value), and color indicates the NES

To explore the transcriptional programs associated with MUCL1 expression, we performed Gene Set Enrichment Analysis (GSEA) in the GSE137356 TNBC dataset, comparing MUCL1-high versus MUCL1-low tumors using the Hallmark gene set collection. MUCL1-high tumors showed significant enrichment of pathways related to androgen response, fatty acid metabolism, xenobiotic metabolism, adipogenesis, and estrogen response (Fig. 2). Additional enriched pathways included mTORC1 signaling, oxidative phosphorylation, epithelial–mesenchymal transition (EMT), and KRAS signaling, indicating a distinct metabolic and signaling profile of MUCL1-high tumors.

### MUCL1 protein expression across histological subtypes and in relation to LAR-associated markers

**Fig. 3:**
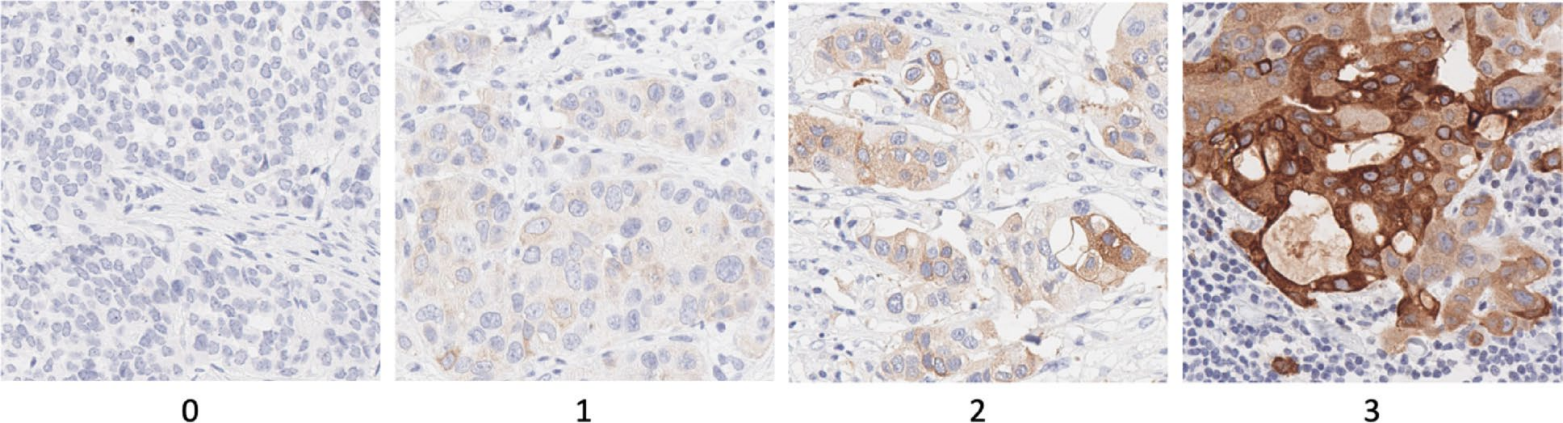
Representative immunohistochemical staining of MUCL1 in breast cancer. Four intensity scores are shown (scores 0–3), corresponding to increasing cytoplasmic staining intensity. Focal membranous accentuation was occasionally observed but was not a consistent feature. Sections are counterstained with hematoxylin. Magnification ×300

Immunohistochemical analysis was performed on 106 TNBC samples representing apocrine, lobular, medullary, metaplastic, and NST subtypes (Table 1). MUCL1 protein expression showed a predominantly cytoplasmic staining pattern with variable intensity across cases, ranging from completely negative (score 0) to strongly positive (score 3). While scoring was based on cytoplasmic intensity, occasional membranous accentuation was observed in tumors with higher expression levels (score 2 and 3). Representative examples of each score are shown in Fig. 3.

**Table 1:** Clinicopathological characteristics of the study cohort

Variable	
*Cohort description*	
Total cases	T stage (including ypT)
106 (100%)	T1: 45 (42.5%)
Age at diagnosis (mean ± SD)	T2: 46 (43.4%)
59.2 ± 15.2	T3: 8 (7.5%)
Histological subtypes	T4: 7 (6.6%)
NST: 74 (69.8%)	Nodal status
Metaplastic: 12 (11.3%)	N+ : 40 (41.7%)
Medullary: 8 (7.5%)	N0: 56 (58.3%)
Apocrine: 6 (5.7%)	Neoadjuvant chemotherapy
Lobular: 6 (5.7%)	Yes: 65 (61.3%)

Summary of clinicopathological parameters in 106 triple-negative breast cancers included in this study. T stage refers to pathological T classification, including ypT categories for patients who received neoadjuvant therapy. Values are presented as number (percentage) or mean ± standard deviation

**Fig. 4:**
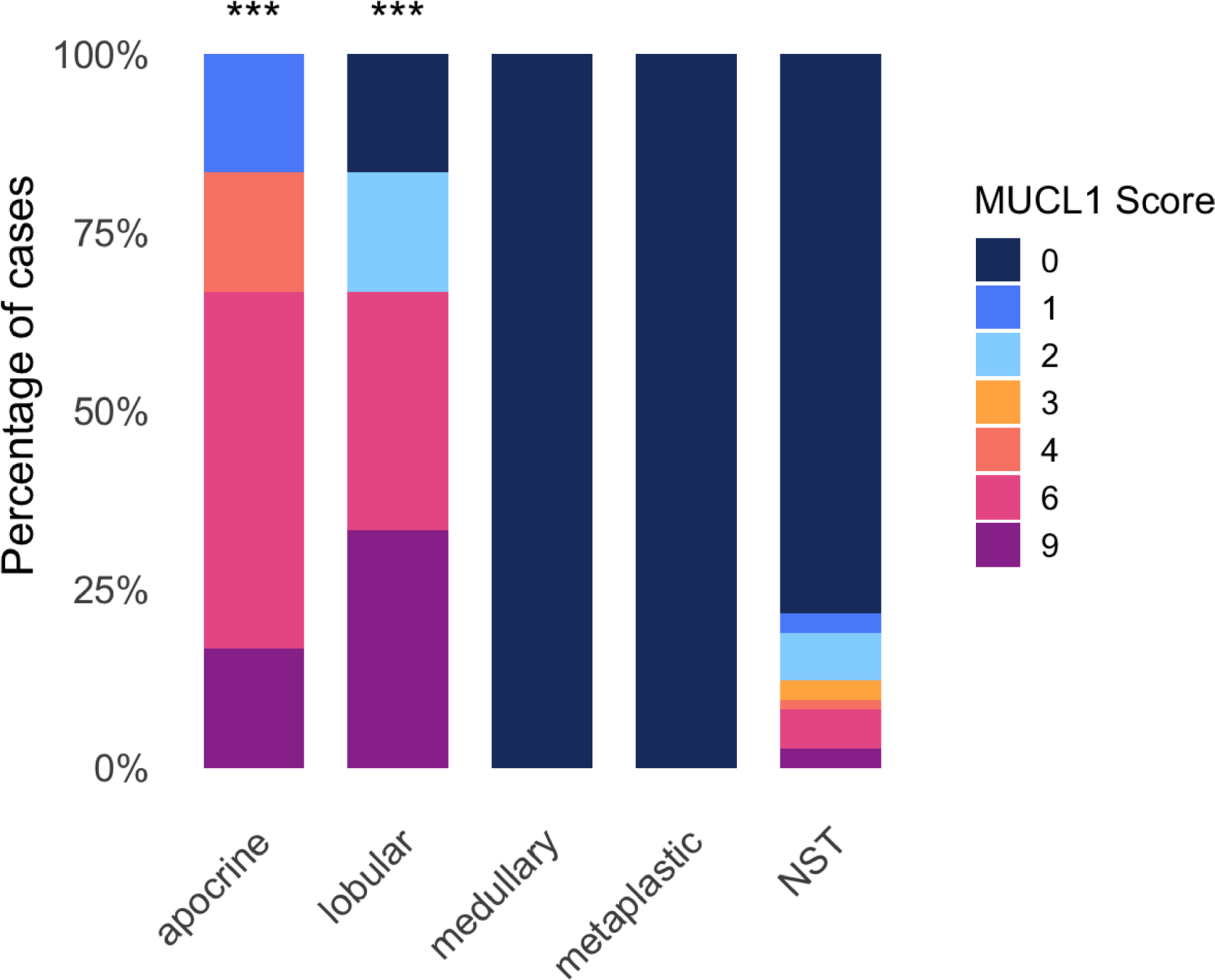
Distribution of MUCL1 scores across histological subtypes of triple-negative breast cancer. Stacked barplot showing the percentage of cases per MUCL1 immunohistochemistry score (0, 1, 2, 3, 4, 6, 9) within each histological subtype. Statistical comparison versus NST subtype was performed using Wilcoxon rank-sum test; significant differences are indicated by asterisks above the bars (***p < 0.001)

To determine the distribution of MUCL1 across histological subtypes and its relation to apocrine differentiation, we next compared MUCL1 staining patterns among distinct TNBC morphologies. As illustrated in the stacked bar plot (Fig. 4), apocrine (6/6; 100%) and lobular carcinomas (5/6; 83%) showed markedly higher rates of MUCL1 positivity compared with NST carcinomas (16/74; 22%). Wilcoxon rank-sum testing confirmed a significant difference for both apocrine vs NST (*p* = 1.4 × 10⁻^5^) and lobular vs NST (*p* = 2.5 × 10⁻^4^). In contrast, medullary and metaplastic carcinomas consistently lacked MUCL1 expression. Statistical comparisons to NST did not reach significance (medullary: *p* = 0.151; metaplastic: *p* = 0.0681).

**Fig. 5:**
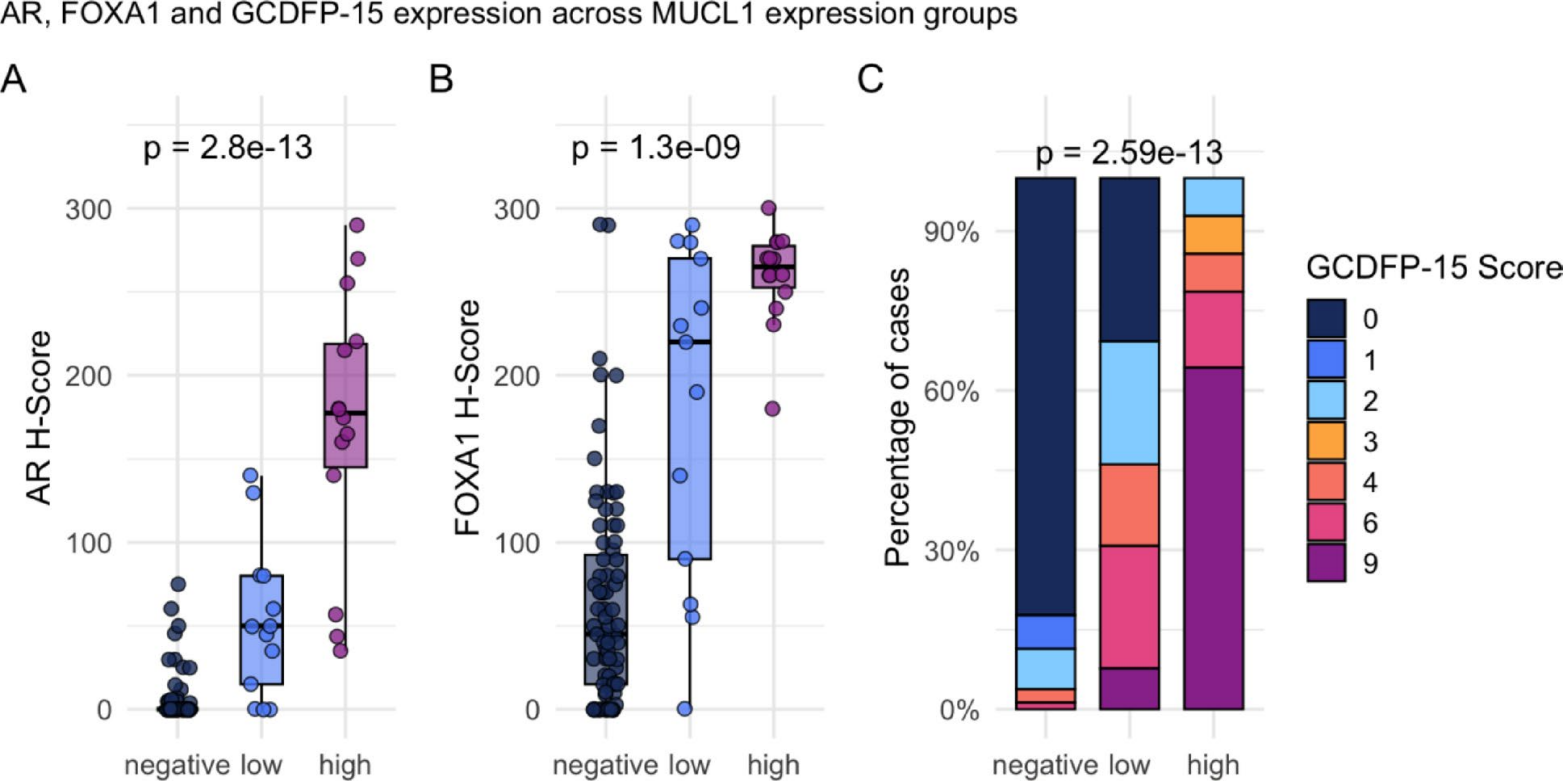
Association of MUCL1 expression with AR, FOXA1, and GCDFP-15 immunohistochemical scores. **A**, **B** Dot-boxplots of AR and FOXA1 H-scores across MUCL1 expression groups (negative, low, high). **C** Stacked barplot of GCDFP-15 immunohistochemical scores (0, 1, 2, 3, 4, 6, 9) across MUCL1 expression groups. Statistical comparisons were performed using Kruskal–Wallis test; unadjusted *p*-values are shown above the plots

To further explore the association of MUCL1 expression with apocrine differentiation, we next analyzed its relationship with established apocrine markers AR, FOXA1, and GCDFP-15. Immunohistochemistry results were analyzed across MUCL1 expression groups (negative, low, high). AR H-scores showed a significant increase with higher MUCL1 expression (Kruskal–Wallis *p* = 2.8 × 10^–13^; Fig. 5A). FOXA1 H-scores were likewise significantly elevated in the MUCL1-high group (Kruskal–Wallis *p* = 1.3 × 10^–9^; Fig. 5B). GCDFP-15 product scores also increased with higher MUCL1 expression (Kruskal–Wallis *p* = 2.59 × 10^–13^; Fig. 5C).

**Fig. 6:**
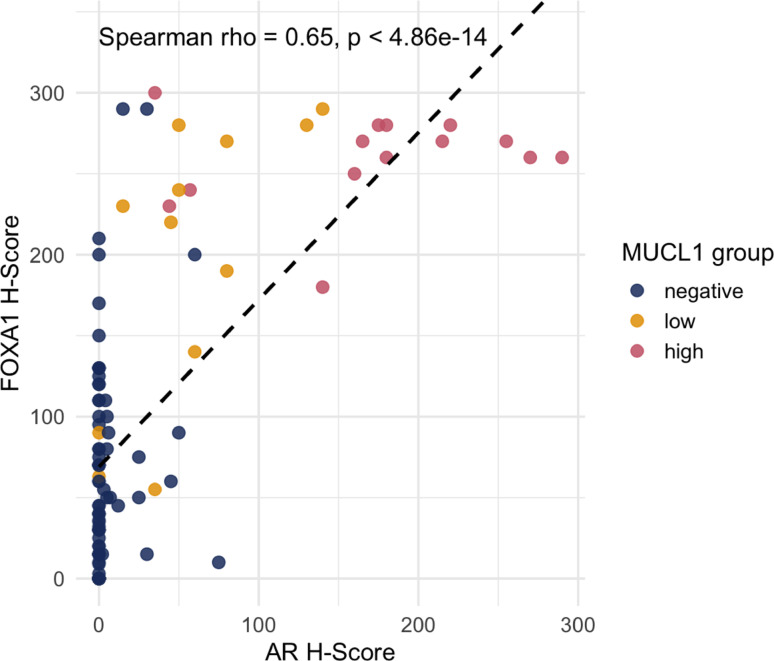
Correlation of AR and FOXA1 H-scores across MUCL1 expression groups. Scatterplot of AR versus FOXA1 H-scores, color-coded by MUCL1 expression group (negative, low, high). A linear regression line (dashed) is shown for visualization. Spearman correlation coefficient (rho) and *p*-value are indicated

AR and FOXA1 H-scores showed a significant positive correlation across the cohort (Spearman’s ρ = 0.62, *p* < 1 × 10^-13^; Fig. 6). Notably, MUCL1-high tumors clustered predominantly in the AR-high and FOXA1-high range (Fig. 6), consistent with the marker profile of LAR-TNBC.

To benchmark MUCL1 against other LAR-associated genes identified in the transcriptomic dataset, we evaluated the protein expression of additional candidates: CLCA2, SPINK8, CYP4F8, and ABCC11, which were significantly upregulated in LAR score-high tumors from GSE137356. While all four genes were consistently upregulated at the transcriptomic level, their protein-level expression in our TNBC cohort revealed variable associations with established apocrine markers. Specifically, CLCA2 and SPINK8 showed significant correlations with AR, FOXA1, and GCDFP-15 protein expression, supporting their potential as surrogate markers of the luminal androgen receptor phenotype. In contrast, CYP4F8 and ABCC11 did not show consistent or statistically significant associations with apocrine differentiation at the protein level. Compared to MUCL1, all of these markers demonstrated weaker and less specific associations at the protein level with established apocrine markers (Supplementary Fig. 1).

### Clinicopathological and molecular features of MUCL1-positive TNBCs

The distribution of clinicopathological parameters by MUCL1/AR group is summarized in Table 2. Patients with MUCL1-positive tumors were significantly older at diagnosis compared to MUCL1–/AR+ and MUCL1–/AR– cases (67.3 vs. 55.9 and 56.5 years, respectively). MUCL1-positive tumors more frequently presented with larger tumor size (T2-T4 in 81.5% vs. 38.9% and 52.5%) and nodal involvement (62.5% vs. 50.0% and 31.0%). They also showed a lower histological grade, with 51.9% of MUCL1-positive tumors being G1/G2 compared to only 16.7% in MUCL1–/AR+ and 16.9% in MUCL1–/AR– cases. Proliferative activity, as measured by the Ki-67 (MIB-1) index and AURKA expression, was lower in MUCL1-positive tumors (mean Ki-67: 12.7%) than in MUCL1–/AR+ (27.8%) and MUCL1–/AR– cases (26.1%). AURKA levels were similarly reduced in the MUCL1-positive group (6.1% vs. 15.0% and 13.4%).

**Table 2:** Summary of clinicopathological parameters according to MUCL1/AR expression group

Variable	MUCL1+	MUCL1-/AR+	MUCL1-/AR-
n	n = 27	n = 18	n = 61
Age at diagnosis (mean ± SD)	67.3 ± 12	55.9 ± 15.5*	56.5 ± 15.3**
T +	22 (81.5%)	7 (38.9%)**	32 (52.5%)*
T1	5 (18.5%)	11 (61.1%)	29 (47.5%)
N +	15 (62.5%)	7 (50%)	18 (31%)*
N0	9 (37.5%)	7 (50%)	40 (69%)
G1/G2	14 (51.9%)	3 (16.7%)	10 (16.9%)
G3	13 (48.1%)	15 (83.3%)*	49 (83.1%)***
Neoadjuvant Chemotherapy	17 (63%)	12 (66.7%)	36 (59%)
Apocrine morphology	6 (22.2%)	0 (0%)	0 (0%)***
Lobular morphology	5 (18.5%)	0 (0%)	1 (1.6%)**
Ki-67 (MIB-1) (mean ± SD)	12.7 ± 21.3	27.8 ± 19.2***	26.1 ± 25.1**
AURKA (mean ± SD)	6.1 ± 7.6	15 ± 12.4**	13.4 ± 11.5**
AR H-score (mean ± SD)	108.6 ± 90.8	22.4 ± 22.1**	0

Values are presented as number (percentage) or mean ± standard deviation

Pairwise comparisons were performed between MUCL1+ and the other two groups (MUCL1–/AR+ and MUCL1–/AR–) using χ^2^ test for categorical variables and Wilcoxon rank-sum test for continuous or ordinal variables

Significant differences versus MUCL1+ are indicated by asterisks (*p < 0.05, **p < 0.01, ***p < 0.001)

Neoadjuvant chemotherapy was administered in a comparable proportion across groups (63% in MUCL1-positive, 66.7% in MUCL1–/AR+ , and 59% in MUCL1–/AR– cases), without a significant difference.

Morphologically, MUCL1-positive tumors were enriched for apocrine and lobular differentiation. Apocrine features were observed in 22.2% of MUCL1-positive tumors, but in none of the MUCL1-negative cases. Similarly, lobular morphology was present in 18.5% of MUCL1-positive tumors, compared to only 1.6% of MUCL1–/AR– tumors and absent in MUCL1–/AR+ cases.

Within AR-positive tumors, MUCL1-positive cases showed substantially higher AR expression levels compared to MUCL1–/AR+ tumors (mean H-score 108.6 vs. 22.4).

Notably, no significant differences were observed between MUCL1-negative/AR-positive and MUCL1-negative/AR-negative tumors across any of the other examined parameters.

To further support this observation, we analyzed AURKA and MKI67 gene expression levels in the GSE137356 TNBC dataset. MUCL1-high tumors showed significantly lower expression of both genes compared to MUCL1-low tumors (Supplementary Fig. 2), indicating an overall reduced proliferative activity in this subgroup.

To more comprehensively illustrate the clinicopathological features across all combinations of MUCL1 and AR status, including the small MUCL1 + /AR − subgroup (n = 3), an extended version of Table 2 is provided in the Supplementary Material (Supplementary Table 1). Despite the limited sample size, this group showed markedly higher proliferative activity, with a mean Ki-67 index of 55% and AURKA expression of 19.3%.

To further characterize the molecular profile associated with MUCL1 expression, we analyzed additional immunohistochemical markers indicative of basal-like and luminal differentiation. MUCL1-positive tumors showed significantly lower SOX10 expression, with 81% of cases being completely negative (score 0) compared to 28% in the MUCL1-negative group (*p* < 0.001). CK18 intensity scores were significantly higher in MUCL1-positive tumors, consistent with a luminal epithelial phenotype (*p* < 0.001). RB1 expression also differed significantly between groups (*p* = 0.033), with MUCL1-negative tumors more frequently exhibiting reduced or absent nuclear RB1 staining (scores 0–1), while MUCL1-positive tumors more often showed strong RB1 expression (score 3, Supplementary Fig. 3).

## Discussion

Triple-negative breast cancer (TNBC) comprises several transcriptionally defined subtypes. Among these, the luminal androgen receptor (LAR) subtype represents approximately 15% of cases and is characterized by luminal gene expression programs and androgen receptor (AR) expression [5, 22]. While LAR-TNBCs often show apocrine or lobular differentiation, morphologically defined apocrine carcinomas and immunohistochemical surrogates (e.g. AR-positive TNBC) only partially overlap with the transcriptomically defined, morphologically heterogeneous LAR subtype, which does not consistently exhibit classic apocrine features. The extent of this overlap depends on the applied morphologic and immunohistochemical definitions, resulting in diagnostic ambiguity [7, 23–26]. LAR-TNBCs often exhibit poor response to chemotherapy, with markedly reduced pathological complete response (pCR) rates compared to non-LAR subtypes [5, 6]. Given the prognostic relevance of pCR [27], a refined molecular definition of LAR-TNBC is critical to improve diagnostic accuracy and guide therapy stratification.

In this study, we aimed to identify novel markers associated with LAR biology using a transcriptome-driven approach. A LAR gene signature was derived from two independent breast cancer datasets (GSE1561 [28] and GSE68892 [29]), comparing LAR-TNBCs to non-LAR subgroups. The overlapping differentially expressed genes were used to construct a 46-gene signature. We subsequently applied this signature to the GSE137356 dataset [30], which includes 381 TNBC cases profiled using a high-density array platform. For each sample, a LAR score was calculated by summing the normalized expression values of positively associated genes and subtracting the values of negatively associated genes.

Among the top differentially expressed genes between high- and low-scoring tumors, MUCL1 (also known as Small Breast Epithelial Mucin, SBEM) emerged as one of the most significantly upregulated candidates in LAR score-high tumors. It encodes a mucin-like glycoprotein with highly restricted expression in breast tissue and breast cancer and only limited expression in other tissues [17]. MUCL1 has been linked to chemotherapy resistance and EMT in colorectal cancer [31], and to poor prognosis and EMT in breast cancer [19, 32]. Despite this, its role in TNBC, and particularly in the LAR subtype, has not been systematically examined. A recent DNA methylation–based study identified MUCL1 as a highly upregulated gene in the non-basal type 2 epitype of TNBC, a subgroup markedly enriched for LAR tumors, providing the first indirect link between MUCL1 and the LAR phenotype [33].

To explore its clinical relevance, we assessed MUCL1 protein expression by immunohistochemistry in a well-annotated retrospective cohort of TNBC cases. MUCL1 expression showed strong correlation with AR, FOXA1, and GCDFP-15 (PIP), consistent with previously described features of the LAR TNBC subtype [15, 25]. In our cohort, MUCL1 expression clustered predominantly within AR-high and FOXA1-high tumors, consistent with prior evidence that AR/FOXA1 co-expression defines a morphologically distinct, luminal-like TNBC subgroup [10, 34]. MUCL1-positive tumors were significantly associated with older age at diagnosis, apocrine and lobular histology, lower histological grade, reduced Ki-67 expression, and a higher frequency of lymph node involvement. These features align with previously reported characteristics of LAR-TNBC and support the biological distinctness of this MUCL1-positive subgroup [6, 22, 25, 35]. Notably, MUCL1-positive tumors also exhibited significantly higher T-stages at resection—an association reported in some studies for MUCL1- or AR-positive tumors [26, 36], though not consistently across the literature [6, 22, 32].

To further clarify the phenotypic distinctness of MUCL1 expression, we compared MUCL1-positive tumors to MUCL1-negative cases stratified by AR status. MUCL1-positive tumors showed significantly larger size, lower grade, and reduced proliferation compared to both MUCL1–/AR+ and MUCL1–/AR– subgroups. Notably, MUCL1–/AR+ tumors more closely resembled MUCL1–/AR– cases than MUCL1-positive ones, suggesting that AR expression alone may not fully capture LAR-associated biological characteristics within our cohort. This underscores ongoing uncertainty surrounding the utility of AR alone as a surrogate for identifying LAR-TNBC, given the lack of consensus on cutoffs and proposals for FOXA1 co-expression or alternative marker panels [10, 25, 37]. At the same time, our data suggest a gradual transition of LAR-associated phenotypic characteristics across increasing MUCL1 expression rather than clearly separable subgroups. In line with this, the presence of a small subset of MUCL1-positive tumors with comparatively low or even absent AR expression further highlights the biological heterogeneity within AR-defined LAR-TNBC and supports a role for MUCL1 in refining this group. Within this context, similar ambiguity exists with regard to morphologically apocrine TNBCs, which are often, but not consistently, AR-positive. While some studies report uniform AR expression in apocrine carcinomas, others describe a substantial proportion of AR-negative cases, depending on the applied diagnostic criteria and methods [7, 26]. MUCL1 may help resolve this diagnostic uncertainty and improve the immunohistochemical identification of LAR-TNBC within this heterogeneous group.

To further support the classification of MUCL1-positive tumors as LAR-TNBCs, we evaluated the expression of CK18, SOX10, and RB1 by immunohistochemistry. MUCL1-positive tumors demonstrated consistently stronger CK18 staining and significantly lower SOX10 expression, reflecting luminal differentiation and reduced basal features, phenotypic traits previously described in LAR- or AR-positive TNBCs [38, 39]. In addition, RB1 expression was more frequently preserved in MUCL1-positive tumors, aligning with prior observations that RB1 loss is less common in LAR-TNBC compared to basal-like subtypes [35, 40]. These findings provide additional immunophenotypic evidence that MUCL1-positive tumors represent a distinct luminal subgroup within TNBC.

GSEA of MUCL1-high tumors in the GSE137356 dataset revealed significant enrichment of androgen response, estrogen response, xenobiotic metabolism, and fatty acid metabolism pathways. These transcriptional programs have been consistently described in LAR-like TNBC [5, 41, 42], further supporting an association between MUCL1 expression and transcriptional programs characteristic of LAR.

Several studies have shown that LAR-TNBCs are characterized by reduced pCR rates after neoadjuvant chemotherapy [5, 6], and in some cohorts this has been associated with less favorable outcomes [43]. In contrast, relatively favorable outcomes have been reported in TNBC with apocrine morphology, raising the possibility of overtreatment with routine neoadjuvant chemotherapy in this subgroup [44, 45]. This may be particularly relevant for AR-positive tumors harboring PI3K pathway alterations, which have been associated with reduced chemotherapy responsiveness in recent studies [46]. These seemingly discordant findings regarding outcome likely reflect differences between morphologically defined apocrine carcinomas and transcriptionally defined LAR subtype, as well as heterogeneity in marker definitions and cohort composition. MUCL1 itself has also been associated with reduced chemotherapy sensitivity in colorectal [31] and breast cancer [18], as well as with adverse outcomes in TNBC [32], and its alignment with LAR-associated transcriptomic programs suggests a potential role in mediating intrinsic chemoresistance. Although we did not assess treatment response directly, these findings support future evaluation of MUCL1 expression in pre-treatment biopsies with known pCR status. If validated, MUCL1 may serve as a biomarker to guide personalized therapy and identify patients unlikely to benefit from standard chemotherapy.

MUCL1 is easily assessable by immunohistochemistry and shows a distinct diffuse cytoplasmic staining pattern. As a glycoprotein with both cytoplasmic expression and extracellular secretion [17], MUCL1 has been discussed as a potential therapeutic target in several publications [17, 31]. Our data suggest that this may be particularly relevant in LAR-TNBCs, where MUCL1 appears to be frequently expressed and may represent a subtype-specific therapeutic target.

In conclusion, MUCL1 is a novel immunohistochemical marker strongly associated with features characteristic of LAR-TNBC, defining a subgroup enriched for apocrine and lobular differentiation, lower proliferative activity, lower histological grade, and transcriptional programs consistent with androgen receptor signaling. Compared to AR alone, MUCL1 identifies a more coherent clinicopathological phenotype aligned with LAR-associated features, suggesting improved discriminatory capacity within this spectrum. While our study does not establish specificity for the molecularly defined LAR subtype, MUCL1 may complement existing immunohistochemical markers such as AR, FOXA1, and GCDFP-15 and improve the approximation of this subtype in routine diagnostic settings. Beyond its diagnostic utility, MUCL1 may also represent a candidate predictive biomarker and potential therapeutic target in this context. Further validation of these findings in cohorts with molecular subtyping is warranted.

## Supplementary Information

Below is the link to the electronic supplementary material.

Supplementary Material 1

Supplementary Material 2

Supplementary Material 3

Supplementary Material 4

## Data Availability

The datasets analyzed during the current study are available in the Gene Expression Omnibus (GEO) repository: https://www.ncbi.nlm.nih.gov/geo/query/acc.cgi?acc=GSE1561, https://www.ncbi.nlm.nih.gov/geo/query/acc.cgi?acc=GSE68892, https://www.ncbi.nlm.nih.gov/geo/query/acc.cgi?acc=GSE137356. All data generated or analyzed during this study are included in this published article and its supplementary information files.

## References

[1] Xiong N, Wu H, Yu Z. Advancements and challenges in triple-negative breast cancer: a comprehensive review of therapeutic and diagnostic strategies. Front Oncol. 2024;14:1405491. 38863622 10.3389/fonc.2024.1405491 PMC11165151

[2] Dent R, et al. Triple-negative breast cancer: clinical features and patterns of recurrence. Clin Cancer Res. 2007;13:4429–34. 17671126 10.1158/1078-0432.CCR-06-3045

[3] Bauer KR, Brown M, Cress RD, Parise CA, Caggiano V. Descriptive analysis of estrogen receptor (ER)‐negative, progesterone receptor (PR)‐negative, and HER2‐negative invasive breast cancer, the so‐called triple‐negative phenotype: a population‐based study from the California cancer Registry. Cancer. 2007;109:1721–8. 17387718 10.1002/cncr.22618

[4] Bianchini G, Balko JM, Mayer IA, Sanders ME, Gianni L. Triple-negative breast cancer: challenges and opportunities of a heterogeneous disease. Nat Rev Clin Oncol. 2016;13:674–90. 27184417 10.1038/nrclinonc.2016.66 PMC5461122

[5] Lehmann BD, et al. Identification of human triple-negative breast cancer subtypes and preclinical models for selection of targeted therapies. J Clin Invest. 2011;121:2750–67. 21633166 10.1172/JCI45014 PMC3127435

[6] Thompson KJ, et al. Luminal androgen receptor breast cancer subtype and investigation of the microenvironment and neoadjuvant chemotherapy response. NAR Cancer. 2022;4:zcac018. 35734391 10.1093/narcan/zcac018 PMC9204893

[7] Nishida H, Kato A, Kaimori R, Kawamura K, Daa T. Relationship between androgen receptor and androgen receptor-related protein expression in breast cancers focusing on morphologically identified carcinoma with apocrine differentiation. Sci Rep. 2025;15:2892. 39843553 10.1038/s41598-025-87403-y PMC11754918

[8] Long M, et al. AR expression correlates with distinctive clinicopathological and genomic features in breast cancer regardless of ESR1 expression status. Int J Mol Sci. 2022;23:11468. 36232774 10.3390/ijms231911468 PMC9570294

[9] Gerratana L, et al. Androgen receptor in triple negative breast cancer: a potential target for the targetless subtype. Cancer Treat Rev. 2018;68:102–10. 29940524 10.1016/j.ctrv.2018.06.005

[10] Guiu S, et al. Prognostic value of androgen receptor and FOXA1 co-expression in non-metastatic triple negative breast cancer and correlation with other biomarkers. Br J Cancer. 2018;119:76–9. 29880907 10.1038/s41416-018-0142-6 PMC6035246

[11] Collins LC, et al. Androgen receptor expression in breast cancer in relation to molecular phenotype: results from the Nurses’ Health Study. Mod Pathol. 2011;24:924–31. 21552212 10.1038/modpathol.2011.54 PMC3128675

[12] Robinson JLL, et al. Androgen receptor driven transcription in molecular apocrine breast cancer is mediated by FoxA1. EMBO J. 2011;30:3019–27. 21701558 10.1038/emboj.2011.216 PMC3160190

[13] Darb-Esfahani S, et al. Gross cystic disease fluid protein 15 (GCDFP-15) expression in breast cancer subtypes. BMC Cancer. 2014;14:546. 25070172 10.1186/1471-2407-14-546 PMC4122770

[14] Loos S, Schulz KD, Hackenberg R. Regulation of GCDFP-15 expression in human mammary cancer cells. Int J Mol Med. 1999;4:135–75. 10402478 10.3892/ijmm.4.2.135

[15] Hu H, et al. Subtyping of triple-negative breast cancers: its prognostication and implications in diagnosis of breast origin. ESMO Open. 2024;9:102993. 38613910 10.1016/j.esmoop.2024.102993 PMC11024544

[16] Habashy HO, et al. Forkhead-box A1 (FOXA1) expression in breast cancer and its prognostic significance. Eur J Cancer. 2008;44:1541–51. 18538561 10.1016/j.ejca.2008.04.020

[17] Conley SJ, et al. HER2 drives Mucin-like 1 to control proliferation in breast cancer cells. Oncogene. 2016;35:4225–34. 26725324 10.1038/onc.2015.487 PMC4996539

[18] Supplitt S, et al. The analysis of transcriptomic signature of TNBC—searching for the potential RNA-based predictive biomarkers to determine the chemotherapy sensitivity. J Appl Genet. 2025;66:171–82. 38722458 10.1007/s13353-024-00876-x PMC11761126

[19] Li Q, et al. Small breast epithelial mucin promotes the invasion and metastasis of breast cancer cells via promoting epithelial-to-mesenchymal transition. Oncol Rep. 2020;44:509–18. 32627029 10.3892/or.2020.7640 PMC7336452

[20] Skliris GP, et al. Expression of small breast epithelial mucin (SBEM) protein in tissue microarrays (TMAs) of primary invasive breast cancers. Histopathology. 2008;52:355–69. 18269587 10.1111/j.1365-2559.2007.02955.x PMC2253716

[21] Xu M, et al. Prognostic significance of Androgen Receptor expression in Triple Negative Breast Cancer: a systematic review and meta-analysis. Clin Breast Cancer. 2020;20:e385–96. 32139270 10.1016/j.clbc.2020.01.002

[22] Lehmann BD, et al. Refinement of triple-negative breast cancer molecular subtypes: implications for neoadjuvant chemotherapy selection. PLoS ONE. 2016;11:e0157368. 27310713 10.1371/journal.pone.0157368 PMC4911051

[23] Quinn CM, D’Arcy C, Wells C. Apocrine lesions of the breast. Virchows Arch. 2022;480:177–89. 34537861 10.1007/s00428-021-03185-4 PMC8983539

[24] Bergeron A, et al. Triple-negative breast lobular carcinoma: a luminal androgen receptor carcinoma with specific ESRRA mutations. Mod Pathol. 2021;34:1282–96. 33753865 10.1038/s41379-021-00742-9 PMC8216909

[25] Rangel N, et al. Surrogate molecular classification of LAR breast cancer in routine workflow. Endocr Relat Cancer. 2025;32:e240316. 40265610 10.1530/ERC-24-0316

[26] Astvatsaturyan K, Yue Y, Walts AE, Bose S. Androgen receptor positive triple negative breast cancer: clinicopathologic, prognostic, and predictive features. PLoS ONE. 2018;13:e0197827. 29883487 10.1371/journal.pone.0197827 PMC5993259

[27] Sharma P, et al. Pathological response and survival in triple-negative breast cancer following neoadjuvant carboplatin plus docetaxel. Clin Cancer Res. 2018;24:5820–9. 30061361 10.1158/1078-0432.CCR-18-0585 PMC6279513

[28] Farmer P, et al. Identification of molecular apocrine breast tumours by microarray analysis. Oncogene. 2005;24:4660–71. 15897907 10.1038/sj.onc.1208561

[29] Doane AS, et al. An estrogen receptor-negative breast cancer subset characterized by a hormonally regulated transcriptional program and response to androgen. Oncogene. 2006;25:3994–4008. 16491124 10.1038/sj.onc.1209415

[30] Sharma P, et al. Validation of the DNA damage immune response signature in patients with triple-negative breast cancer from the SWOG 9313c trial. J Clin Oncol. 2019;37:3484–92. 31657982 10.1200/JCO.19.00693 PMC7194448

[31] Abdulla M, et al. Targeting MUCL1 protein inhibits cell proliferation and EMT by deregulating β‑catenin and increases irinotecan sensitivity in colorectal cancer. Int J Oncol. 2022. 10.3892/ijo.2022.5312. 35059735 10.3892/ijo.2022.5312 PMC8857929

[32] Liu L, et al. Small breast epithelial mucin tumor tissue expression is associated with increased risk of recurrence and death in triple-negative breast cancer patients. Diagn Pathol. 2013;8:71. 23635316 10.1186/1746-1596-8-71 PMC3680073

[33] Aine M, et al. The DNA methylation landscape of primary triple-negative breast cancer. Nat Commun. 2025;16:3041. 40155623 10.1038/s41467-025-58158-x PMC11953470

[34] Guiu S, et al. Coexpression of androgen receptor and FOXA1 in nonmetastatic triple-negative breast cancer: ancillary study from PACS08 trial. Future Oncol. 2015;11:2283–97. 26260807 10.2217/fon.15.102

[35] Lehmann BD, et al. Multi-omics analysis identifies therapeutic vulnerabilities in triple-negative breast cancer subtypes. Nat Commun. 2021;12:6276. 34725325 10.1038/s41467-021-26502-6 PMC8560912

[36] Liu Z-Z, Xie X-D, Qu S-X, Zheng Z-D, Wang Y-K. Small breast epithelial mucin (SBEM) has the potential to be a marker for predicting hematogenous micrometastasis and response to neoadjuvant chemotherapy in breast cancer. Clin Exp Metastasis. 2010;27:251–9. 20364301 10.1007/s10585-010-9323-2

[37] Bhattarai S, Saini G, Gogineni K, Aneja R. Quadruple-negative breast cancer: novel implications for a new disease. Breast Cancer Res. 2020;22:127. 33213491 10.1186/s13058-020-01369-5 PMC7678108

[38] Coussy F, et al. Response to mTOR and PI3K inhibitors in enzalutamide-resistant luminal androgen receptor triple-negative breast cancer patient-derived xenografts. Theranostics. 2020;10:1531–43. 32042320 10.7150/thno.36182 PMC6993232

[39] Harbhajanka A, et al. Clinicopathological, immunohistochemical and molecular correlation of neural crest transcription factor SOX10 expression in triple-negative breast carcinoma. Hum Pathol. 2018;80:163–9. 29894722 10.1016/j.humpath.2018.06.007 PMC8126991

[40] Jiang Y-Z, et al. Genomic and transcriptomic landscape of triple-negative breast cancers: subtypes and treatment strategies. Cancer Cell. 2019;35:428-440.e5. 30853353 10.1016/j.ccell.2019.02.001

[41] Lee M, et al. Luminal androgen receptor subtype and tumor-infiltrating lymphocytes groups based on triple-negative breast cancer molecular subclassification. Sci Rep. 2024;14:11278. 38760384 10.1038/s41598-024-61640-z PMC11101432

[42] Wang X, et al. Spatial transcriptomics reveals substantial heterogeneity in triple-negative breast cancer with potential clinical implications. Nat Commun. 2024;15:10232. 39592577 10.1038/s41467-024-54145-w PMC11599601

[43] Hartung C, et al. Identifying high-risk triple-negative breast cancer patients by molecular subtyping. Breast Care. 2021;16:637–47. 35082572 10.1159/000519255 PMC8740062

[44] Schwartz CJ, et al. Triple-negative apocrine carcinomas: toward a unified group with shared molecular features and clinical behavior. Mod Pathol. 2023;36:100125. 36870308 10.1016/j.modpat.2023.100125

[45] Srivastava P, et al. Clinical-pathologic characteristics and response to neoadjuvant chemotherapy in triple-negative low Ki-67 proliferation (TNLP) breast cancers. Npj Breast Cancer. 2022;8:51. 35444182 10.1038/s41523-022-00415-z PMC9021249

[46] Basho RK, et al. Comprehensive analysis identifies variability in PI3K pathway alterations in triple-negative breast cancer subtypes. JCO Precis Oncol. 2024. 10.1200/PO.23.00124. 38484209 10.1200/PO.23.00124 PMC10954064

