# Supplementary material for: MUCL1 in triple-negative breast cancer: a novel marker associated with the luminal androgen receptor subtype
Source: Breast Cancer Res. 2026 Apr 15;28:73. doi: 10.1186/s13058-026-02283-y (PMC13088539; doi:10.1186/s13058-026-02283-y)
Supplement: Supplementary file 4 — Supplementary Material 4 [file 13058_2026_2283_MOESM4_ESM.docx]

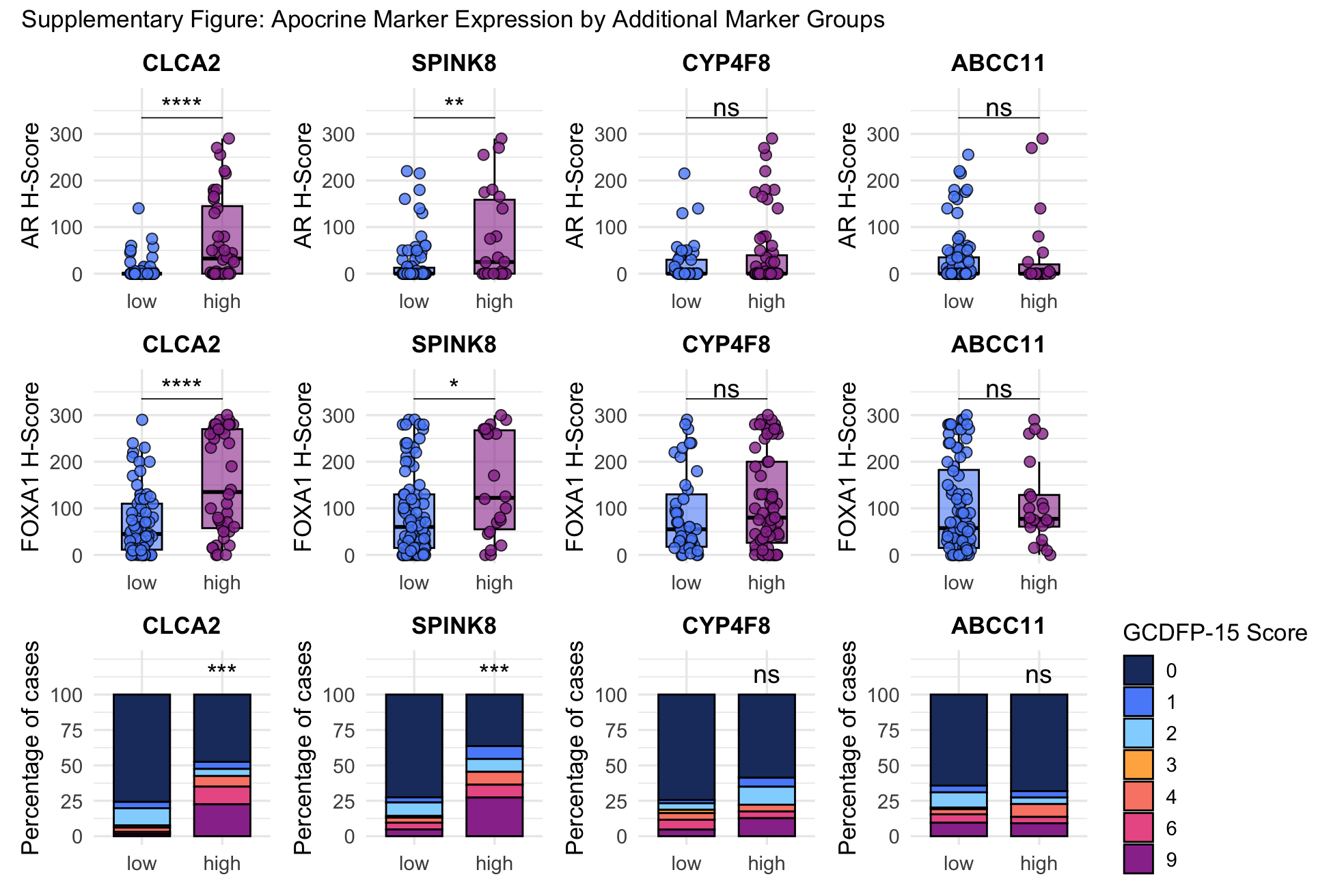


**Supplementary Figure 1:** Expression of AR, FOXA1, and GCDFP-15 across tumors stratified by CLCA2, SPINK8, CYP4F8, and ABCC11 expression (low vs. high). Boxplots show H-scores for AR and FOXA1; bar plots depict the distribution of GCDFP-15 scores. P-values were calculated using Wilcoxon tests.


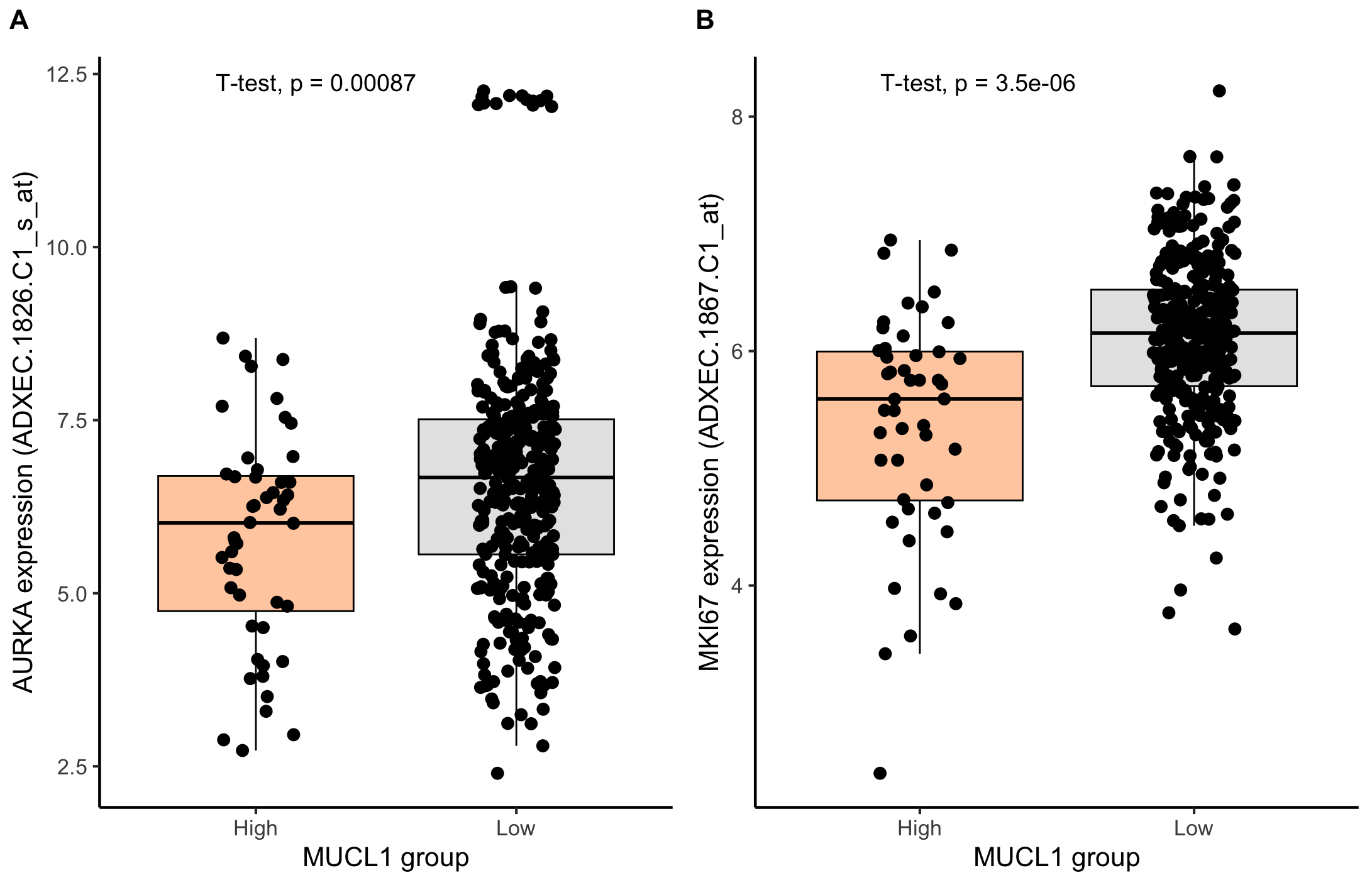


**Supplementary Figure 2** Association of MUCL1 expression with AURKA and MKI67 gene expression in GSE137356 TNBC dataset. Bar and dotplots show (A) AURKA (probe ID ADXEC.1826.C1_s_at) and (B) MKI67 (probe ID ADXEC.1867.C1_at) expression levels in MUCL1 high versus low tumors (cutoff = 7.5). P-values were calculated using t-tests.


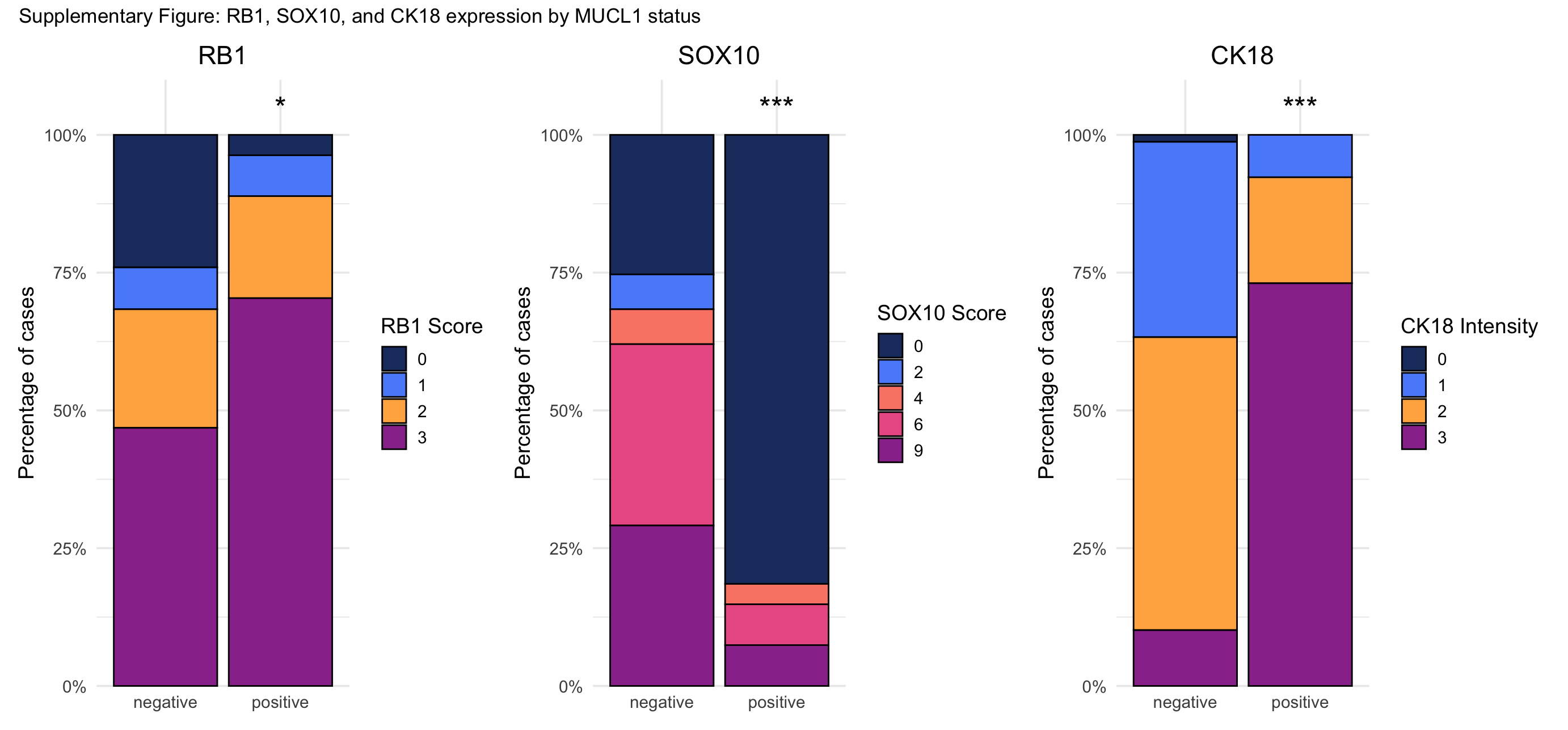


**Supplementary Figure 3.** RB1, SOX10, and CK18 expression in MUCL1-negative vs. MUCL1-positive TNBCs. Stacked barplots display the distribution of RB1 nuclear staining scores (left), SOX10 expression scores (middle), and CK18 intensity scores (right). Asterisks indicate statistical significance based on Wilcoxon testing (*p < 0.05; ***p < 0.001).


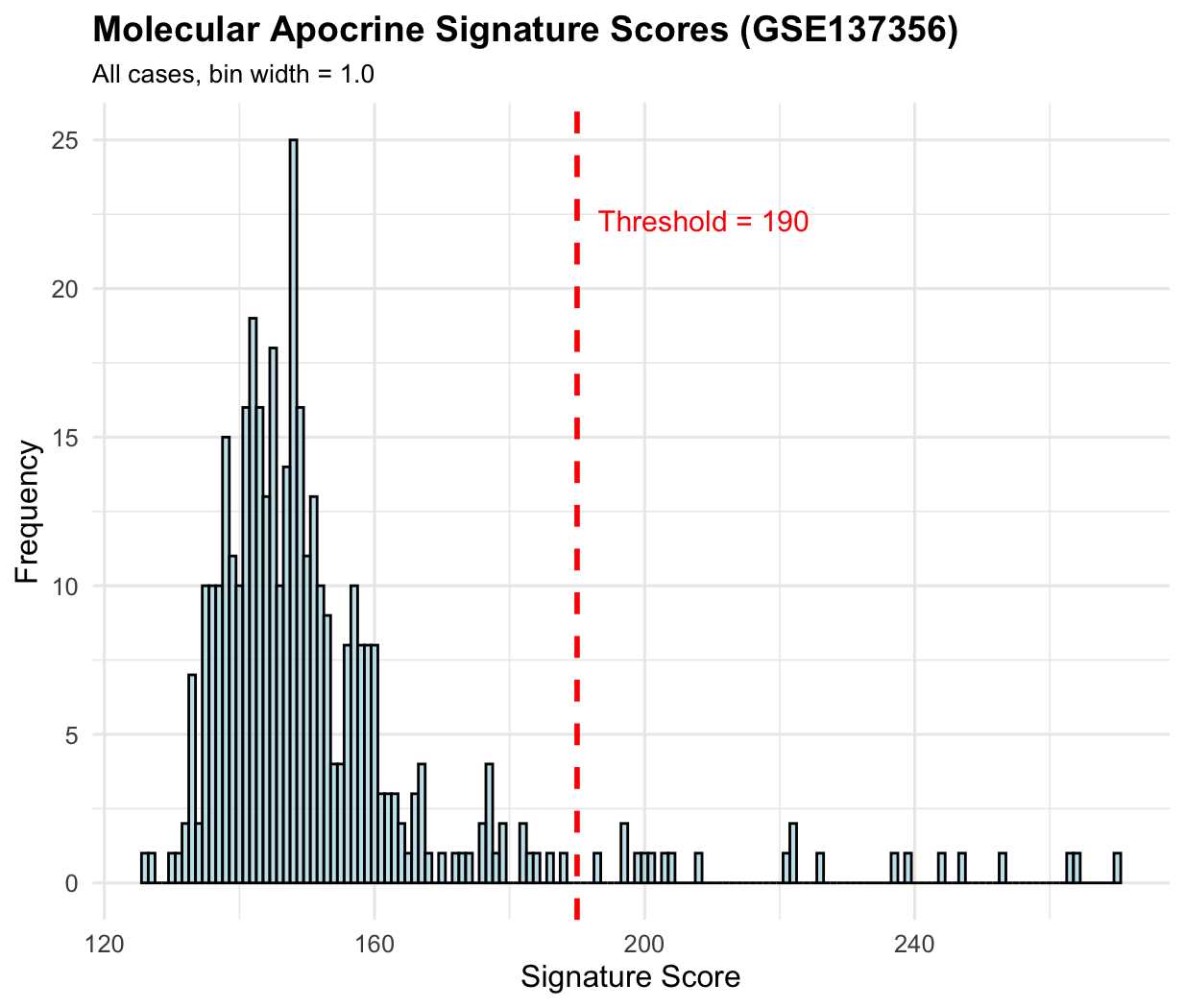


**Supplementary Figure 4**: **Distribution of Luminal Androgen Receptor (LAR) Signature Scores in GSE137356.**
Histogram showing the distribution of LAR signature scores across all TNBC samples (n = 381) in the GSE137356 dataset. A bin width of 1.0 was used. The red dashed line indicates the empirically defined threshold of 190, used to stratify tumors into LAR score-high and -low groups.

Summary of clinicopathological parameters according to MUCL1/AR group

| **Variable** | **MUCL1+/AR+** | **MUCL1+/AR−** | **MUCL1−/AR+** | **MUCL1−/AR−** |
| --- | --- | --- | --- | --- |
| n | n = 24 | n = 3 | n = 18 | n = 61 |
| Age at diagnosis (mean ± SD) | 66.8 ± 12.1 | 71 ± 12.5 | 55.9 ± 15.5 | 56.5 ± 15.3 |
| T+ | 20 (83.3%) | 2 (66.7%) | 7 (38.9%) | 32 (52.5%) |
| T1 | 4 (16.7%) | 1 (33.3%) | 11 (61.1%) | 29 (47.5%) |
| N+ | 13 (61.9%) | 2 (66.7%) | 7 (50%) | 18 (31%) |
| N0 | 8 (38.1%) | 1 (33.3%) | 7 (50%) | 40 (69%) |
| G1/G2 | 14 (58.3%) | 0 (0%) | 3 (16.7%) | 10 (16.9%) |
| G3 | 10 (41.7%) | 3 (100%) | 15 (83.3%) | 49 (83.1%) |
| Neoadjuvant chemotherapy | 15 (62.5%) | 2 (66.7%) | 12 (66.7%) | 36 (59%) |
| Apocrine morphology | 6 (25.0%) | 0 (0%) | 0 (0%) | 0 (0%) |
| Lobular morphology | 5 (20.8%) | 0 (0%) | 0 (0%) | 1 (1.6%) |
| Ki-67 (MIB-1) (mean ± SD) | 7.4 ± 11.9 | 55 ± 35 | 27.8 ± 19.2 | 26.1 ± 25.1 |
| AURKA (mean ± SD) | 4.4 ± 5.1 | 19.3 ± 12.2 | 15 ± 12.4 | 13.4 ± 11.5 |
| AR H-score | 122.2 ± 85.1 | 0 ± 0 | 22.4 ± 22.1 | 0 ± 0 |

**Supplementary Table 1:** Extended summary of clinicopathological parameters across all MUCL1/AR subgroups in triple-negative breast cancer.
This table includes the additional subgroup of MUCL1+/AR− tumors (n = 3), which was omitted from the main Table 1 due to small sample size. Values are presented as number (percentage) or mean ± standard deviation.
